# Supplementary material for: Leishmania Animal Models Used in Drug Discovery: A Systematic Review
Source: Animals (Basel). 2023 May 16;13(10):1650. doi: 10.3390/ani13101650 (PMC10215483; doi:10.3390/ani13101650)
Supplement: Supplementary file 1 [file animals-13-01650-s001.zip › animals-2368557-supplementary.pdf]

Supplementary Document S1 - SEARCH STRATEGIES

The data bases PUBMED, EMBASE, LILACS and SCIELO were last searched on March 29, 2020. The complete search syntaxes are presented below.

PUBMED

Website: **https://pubmed.ncbi.nlm.nih.gov**

Applied search (29 March 2020 – resulted in retrieval of 5469 records):
(leishman* [tiab] AND (Donovani [tiab] OR infantum[tiab] OR chagasi [tiab] OR mexicana [tiab] OR amazonensis [tiab] OR major [tiab] OR tropica [tiab] OR braziliens* [tiab] OR brasiliens* [tiab] OR guyanens* [tiab] OR panamens* [tiab])) OR Delhi boil [tiab] OR assam fever [tiab] OR Oriental sore [tiab] OR kala azar [tiab] OR kala-azar [tiab] OR tropical sore [tiab] OR Aleppo boil [tiab] OR dumdum fever [tiab] OR black fever [tiab] OR bos yaws [tiab] OR pian bois [tiab] AND Therapeutics[Mesh] OR therapy [tiab] OR therapies [tiab] OR therapeutic [tiab] OR therapeutics [tiab] OR medicine [tiab] OR drug [tiab] OR drugs [tiab] OR treatment [tiab] OR treatments [tiab] OR compound [tiab] OR compounds [tiab] OR pharmacotherapies [tiab] OR pharmacotherapy [tiab]
+ Application of specific Animal filter for PUBMED searches [17]

Excerpta Medica Database (EMBASE)

Website: **www.embase.com**

Applied search (29 March 2020 – resulted in retrieval of 4235 records):
((leishman* and (Donovani or infantum or chagasi or mexicana or amazonensis or major or tropica or braziliens* or brasiliens* or guyanens* or panamens*)) or Delhi boil or assam fever or Oriental sore or kala azar or kala-azar or tropical sore or Aleppo boil or dumdum fever or black fever or bos yaws or pian bois).ab,ti. AND therapeutics.ab. or therapeutics.ti. or (therapy or therapies or therapeutic or therapeutics or medicine or drug or drugs or treatment or treatments or compound or compounds or pharmacotherapies or pharmacotherapy).ab,ti.
+ Application of specific Animal filter for EMBASE searches [16]

Latin American and the Caribbean Health Sciences Literature (LILACS)

Website: http://**lilacs.bvsalud.org**

Applied search (29 March 2020 – resulted in retrieval of 1206 records):
(leishman$ AND (Donovani OR infantum OR chagasi OR mexicana OR amazonensis OR major OR tropica OR braziliens$ OR brasiliens$ OR guyanens$ OR panamens$)) OR Delhi boil OR assam fever OR Oriental sore OR kala azar OR kala-adar OR tropical sore OR Aleppo boil OR dumdum fever OR black fever OR bos yaws OR pian bois

AND
therap$ OR terap$ OR medicin$ OR drug$ OR drog$ OR treatment$ OR tratamient$ OR compound$ OR pharmacotherap$ OR farmacoterap$

Scientific Electronic Library Online (SCIELO)

Website : https://www.scielo.br

Applied search (29 March 2020 – resulted in retrieval of 41 records):
((donovani) OR (infantum) OR (chagasi) OR (mexicana) OR (amazonensis) OR (major) OR (tropica) OR (braziliens*) OR (brasiliens*) OR (guyanens*) OR (panamens*) AND leishman* and (delhi boil) OR (assam fever) OR (oriental sore) OR (kala azar) OR (kala-azar) OR (tropical sore) OR (aleppo boil) OR (dumdum fever) OR (black fever) OR (bos yaws) OR (pian bois)) AND ((therap*) OR (medicine) OR (drug) OR (drugs) OR (treatment) OR (treatments) OR (compound) OR (compounds) OR (pharmacotherapies) OR (pharmacotherapy))

Supplementary Document S2 – LIST OF INCLUDED PAPERS FOR FULL ANALYSIS

The list below provides the bibliographic details of all 203 papers included in this systematic review for data extraction and analysis.

1. Abu Ammar, A., et al. (2019). "Amphotericin B-loaded nanoparticles for local treatment of cutaneous leishmaniasis." Drug Deliv Transl Res 9(1): 76-84.
2. Afrin, F., et al. (2019). "Cinnamomum cassia exhibits antileishmanial activity against Leishmania donovani infection in vitro and in vivo." PLoS Negl Trop Dis 13(5): e0007227.
3. Aguiar, M. G., et al. (2010). "Reductions in skin and systemic parasite burdens as a combined effect of topical paromomycin and oral miltefosine treatment of mice experimentally infected with Leishmania (Leishmania) amazonensis." Antimicrob Agents Chemother 54(11): 4699-4704.
4. Alawa, J. N., et al. (2012). "Infectivity of macrophages and the histopathology of cutaneous lesions, liver and spleen is attenuated by leaf extract of Vernonia amygdalina in Leishmania major infected BALB/c mice." J Complement Integr Med 9: Article 10.
5. Alkathiri, B., et al. (2017). "Pomegranate (Punica granatum) Juice Shows Antioxidant Activity against Cutaneous Leishmaniasis-Induced Oxidative Stress in Female BALB/c Mice." Int J Environ Res Public Health 14(12).
6. Almeida-Souza, F., et al. (2016). "Morinda citrifolia Linn. Reduces Parasite Load and Modulates Cytokines and Extracellular Matrix Proteins in C57BL/6 Mice Infected with Leishmania (Leishmania) amazonensis." PLoS Negl Trop Dis 10(8): e0004900.
7. Amer, E. I., et al. (2016). "Oral azithromycin versus its combination with miltefosine for the treatment of experimental Old World cutaneous leishmaniasis." Journal of Parasitic Diseases 40(2): 475-484.
8. Antinarelli, L. M. R., et al. (2018). "Antileishmanial activity of a 4-hydrazinoquinoline derivative: Induction of autophagy and apoptosis-related processes and effectiveness in experimental cutaneous leishmaniasis." Exp Parasitol 195: 78-86.
9. Anversa, L., et al. (2017). "Amiodarone and itraconazole improve the activity of pentavalent antimonial in the treatment of experimental cutaneous leishmaniasis." Int J Antimicrob Agents 50(2): 159-165.
10. Aoki, J. I., et al. (2011). "Efficacy of the tubercidin anti Leishmania action associated with an inhibitor of the nucleoside transport." Tropical Medicine and International Health 1: 222-223.
11. Aragao Macedo, S. R., et al. (2019). "Evaluation of the antileishmanial activity of biodegradable microparticles containing a hexanic eluate subfraction of Maytenus guianensis bark." Exp Parasitol 205: 107738.
12. Araujo, I. A. C., et al. (2019). "Efficacy of lapachol on treatment of cutaneous and visceral leishmaniasis." Exp Parasitol 199: 67-73.
13. Arruda-Costa, N., et al. (2017). "Anti-parasitic effect of the diuretic and Na+-ATPAse inhibitor furosemide in cutaneous leishmaniasis." Parasitology 144(10): 1375-1383.
14. Azevedo, E. G., et al. (2014). "Mixed formulation of conventional and pegylated liposomes as a novel drug delivery strategy for improved treatment of visceral leishmaniasis." Expert Opin Drug Deliv 11(10): 1551-1560.
15. Bahrami, S., et al. (2015). "Potential application of nanochitosan film as a therapeutic agent against cutaneous leishmaniasis caused by L. major." Parasitol Res 114(12): 4617-4624.
16. Balana-Fouce, R., et al. (2012). "Indotecan (LMP400) and AM13-55: two novel indenoisoquinolines show potential for treating visceral leishmaniasis." Antimicrob Agents Chemother 56(10): 5264-5270.
17. Barao, S. C. and S. Giorgio (2003). "Efficacy of 8-bromoguanosine against murine cutaneous leishmaniasis induced with Leishmania amazonensis." Chemotherapy 49(4): 159-162.

18. Barros, N. B., et al. (2013). "Liposomal-lupane system as alternative chemotherapy against cutaneous leishmaniasis: macrophage as target cell." Exp Parasitol 135(2): 337-343.
19. Bemani, E., et al. (2019). "Effectiveness of amiodarone in treatment of cutaneous leishmaniasis caused by Leishmania major." Exp Parasitol 205: 107747.
20. Bhaumik, S. K., et al. (2012). "Asiaticoside induces tumour-necrosis-factor-alpha-mediated nitric oxide production to cure experimental visceral leishmaniasis caused by antimony-susceptible and -resistant Leishmania donovani strains." J Antimicrob Chemother 67(4): 910-920.
21. Borazjani, R., et al. (2018). "Effect of hydroalcoholic extract of arnebia euchroma on the treatment of cutaneous leishmaniasis." Journal of Clinical and Diagnostic Research 12(8): DC21-DC23.
22. Brito, S., et al. (2006). "[Efficacy of a kaurenic acid extracted from the Venezuelan plant Wedelia trilobata (Asteracea) against Leishmania (Viannia) braziliensis]." Biomedica 26: 180-187.
23. Carregal, V. M., et al. (2019). "Combination oral therapy against Leishmania amazonensis infection in BALB/c mice using nanoassemblies made from amphiphilic antimony(V) complex incorporating miltefosine." Parasitol Res 118(10): 3077-3084.
24. Carter, K. C., et al. (2003). "The in vivo susceptibility of Leishmania donovani to sodium stibogluconate is drug specific and can be reversed by inhibiting glutathione biosynthesis." Antimicrob Agents Chemother 47(5): 1529-1535.
25. Carvalheiro, M., et al. (2015). "Hemisynthetic trifluralin analogues incorporated in liposomes for the treatment of leishmanial infections." Eur J Pharm Biopharm 93: 346-352.
26. Casa, D. M., et al. (2018). "Bovine serum albumin nanoparticles containing amphotericin B were effective in treating murine cutaneous leishmaniasis and reduced the drug toxicity." Exp Parasitol 192: 12-18.
27. Castro, R. A., et al. (2014). "Association of liposome-encapsulated trivalent antimonial with ascorbic acid: an effective and safe strategy in the treatment of experimental visceral leishmaniasis." PLoS One 9(8): e104055.
28. Charret, K. S., et al. (2009). "Effect of oral treatment with pyrazole carbohydrazide derivatives against murine infection by Leishmania amazonensis." Am J Trop Med Hyg 80(4): 568-573.
29. Chegeni, A. S., et al. (2016). "Effect of peel and leaf extract of walnut (Juglans Regia L.) on cutaneous leishmaniasis caused by leishmania major in BALB/c Mice." Journal of Chemical and Pharmaceutical Sciences 9(4): 2490-2495.
30. Chowdhury, S. R., et al. (2017). "Voacamine alters Leishmania ultrastructure and kills parasite by poisoning unusual bi-subunit topoisomerase IB." Biochem Pharmacol 138: 19-30.
31. Coelho, A. C., et al. (2016). "A Luciferase-Expressing Leishmania braziliensis Line That Leads to Sustained Skin Lesions in BALB/c Mice and Allows Monitoring of Miltefosine Treatment Outcome." PLoS Negl Trop Dis 10(5): e0004660.
32. Corpas-Lopez, V., et al. (2019). "A nanodelivered Vorinostat derivative is a promising oral compound for the treatment of visceral leishmaniasis." Pharmacol Res 139: 375-383.
33. Corpas-Lopez, V., et al. (2016). "Topical Treatment of Leishmania tropica Infection Using (-)-alpha-Bisabolol Ointment in a Hamster Model: Effectiveness and Safety Assessment." J Nat Prod 79(9): 2403-2407.
34. Corpas-Lopez, V., et al. (2015). "(-)-alpha-Bisabolol, a Promising Oral Compound for the Treatment of Visceral Leishmaniasis." J Nat Prod 78(6): 1202-1207.
35. Corral, M. J., et al. (2014). "Efficacy of low doses of amphotericin B plus allicin against experimental visceral leishmaniasis." J Antimicrob Chemother 69(12): 3268-3274.
36. Correa, E., et al. (2014). "Leishmanicidal and trypanocidal activity of Sapindus saponaria^ien Actividad leishmanicida y tripanocida de Sapindus saponaria^ies." Bol. latinoam. Caribe plantas med. aromát 13(4): 311-323.
37. Costa Duarte, M., et al. (2016). "An effective in vitro and in vivo antileishmanial activity and mechanism of action of 8-hydroxyquinoline against Leishmania species causing visceral and tegumentary leishmaniasis." Vet Parasitol 217: 81-88.
38. Costa, I. S., et al. (2013). "S-nitrosoglutathione (GSNO) is cytotoxic to intracellular amastigotes and promotes healing of topically treated Leishmania major or Leishmania braziliensis skin lesions." J Antimicrob Chemother 68(11): 2561-2568.

39. Costa, L., et al. (2014). "Pterocarpanquinone LQB-118 induces apoptosis in Leishmania (Viannia) braziliensis and controls lesions in infected hamsters." PLoS One 9(10): e109672.
40. Cunha-Junior, E. F., et al. (2016). "Preclinical Studies Evaluating Subacute Toxicity and Therapeutic Efficacy of LQB-118 in Experimental Visceral Leishmaniasis." Antimicrob Agents Chemother 60(6): 3794-3801.
41. da Matta, C. B., et al. (2015). "Novel dialkylphosphorylhydrazones: Synthesis, leishmanicidal evaluation and theoretical investigation of the proposed mechanism of action." Eur J Med Chem 101: 1-12.
42. de Almeida, L., et al. (2017). "In vivo antileishmanial activity and histopathological evaluation in Leishmania infantum infected hamsters after treatment with a furoxan derivative." Biomed Pharmacother 95: 536-547.
43. de Barros, N. B., et al. (2018). "ASP49-phospholipase A<inf>2</inf>-loaded liposomes as experimental therapy in cutaneous leishmaniasis model." International Immunopharmacology 55: 128-132.
44. de Lima, S. C., et al. (2014). "In vitro and in vivo leishmanicidal activity of Astronium fraxinifolium (Schott) and Plectranthus amboinicus (Lour.) Spreng against Leishmania (Viannia) braziliensis." Biomed Res Int 2014: 848293.
45. de Mello, T. F., et al. (2015). "Activity of synthetic chalcones in hamsters experimentally infected with Leishmania (Viannia) braziliensis." Parasitol Res 114(10): 3587-3600.
46. de Morais, S. M., et al. (2014). "Thymol and eugenol derivatives as potential antileishmanial agents." Bioorg Med Chem 22(21): 6250-6255.
47. de Morais-Teixeira, E., et al. (2019). "In vitro activity and in vivo efficacy of fexinidazole against New World Leishmania species." The Journal of antimicrobial chemotherapy 74(8): 2318-2325.
48. de Souza, R. M., et al. (2020). "Lipid nanoparticles for amphotericin delivery in the treatment of American tegumentary leishmaniasis." Drug Delivery and Translational Research 10(2): 403-412.
49. Dea-Ayuela, M. A., et al. (2009). "In vivo and in vitro anti-leishmanial activities of 4-nitro-N-pyrimidin- and N-pyrazin-2-ylbenzenesulfonamides, and N2-(4-nitrophenyl)-N1-propylglycinamide." Bioorg Med Chem 17(21): 7449-7456.
50. Demarchi, I. G., et al. (2012). "Effect of HIV protease inhibitors on New World Leishmania." Parasitol Int 61(4): 538-544.
51. do Espirito Santo, R. D., et al. (2019). "N, N', N''-trisubstituted guanidines: Synthesis, characterization and evaluation of their leishmanicidal activity." Eur J Med Chem 171: 116-128.
52. dos Santos, A. O., et al. (2011). "Leishmania amazonensis: effects of oral treatment with copaiba oil in mice." Exp Parasitol 129(2): 145-151.
53. dos Santos, I. B., et al. (2018). "Leishmanicidal and immunomodulatory activities of the palladacycle complex DPPE 1.1, a potential candidate for treatment of cutaneous leishmaniasis." Frontiers in Microbiology 9(1427).
54. Duarte, M. C., et al. (2016). "Treatment of murine visceral leishmaniasis using an 8-hydroxyquinoline-containing polymeric micelle system." Parasitol Int 65(6): 728-736.
55. Eissa, M. M., et al. (2012). "Miltefosine for Old World cutaneous leishmaniasis: An experimental study on Leishmania major infected mice." Alexandria Journal of Medicine 48(3): 261-271.
56. Escrivani, D. O., et al. (2020). "Encapsulation in lipid-core nanocapsules improves topical treatment with the potent antileishmanial compound CH8." Nanomedicine: Nanotechnology, Biology, and Medicine 24(102121).
57. Espuri, P. F., et al. (2019). "Synthesis and evaluation of the antileishmanial activity of silver compounds containing imidazolidine-2-thione." Journal of Biological Inorganic Chemistry 24(3): 419-432.
58. Fernandes, F. R., et al. (2013). "Amphiphilic antimony(V) complexes for oral treatment of visceral leishmaniasis." Antimicrobial Agents and Chemotherapy 57(9): 4229-4236.
59. Ferreira, F. M., et al. (2014). "Association of water extract of green propolis and liposomal meglumine antimoniate in the treatment of experimental visceral leishmaniasis." Parasitol Res 113(2): 533-543.

60. Freitas, J. C. C., et al. (2011). "Effect of ethyl acetate extract from husk fiber water of Cocos nucifera in Leishmania braziliensis infected hamsters^ien." Rev. bras. farmacogn 21(6): 1006-1011.
61. Garcia Bustos, M. F., et al. (2014). "In vivo antileishmanial efficacy of miltefosine against Leishmania (Leishmania) amazonensis." J Parasitol 100(6): 840-847.
62. Gervazoni, L. F. O., et al. (2018). "2'-Hydroxyflavanone activity in vitro and in vivo against wild-type and antimony-resistant Leishmania amazonensis." PLoS Negl Trop Dis 12(12): e0006930.
63. Ghaffarifar, F., et al. (2015). "Evaluation of apoptotic and antileishmanial activities of artemisinin on promastigotes and BALB/C mice infected with Leishmania major." Iranian Journal of Parasitology 10(2): 258-267.
64. Ghasemi, E., et al. (2019). "In-vitro and in-vivo antileishmanial activity of a compound derived of platinum, oxaliplatin, against leishmania major." Iranian Journal of Pharmaceutical Research 18(4): 2028-2041.
65. Ghosh, P., et al. (2016). "Preparation and characterization of andrographolide nanoparticles for visceral leishmaniasis chemotherapy: In vitro and in vivo evaluations." International Journal of Pharmacy and Pharmaceutical Sciences 8(12): 102-107.
66. Ghosh, S., et al. (2016). "Oleanolic acid loaded poly lactic co- glycolic acid- vitamin E TPGS nanoparticles for the treatment of Leishmania donovani infected visceral leishmaniasis." Int J Biol Macromol 93: 961-970.
67. Godinho, J. L. P., et al. "Efficacy of miltefosine treatment in Leishmania amazonensis-infected BALB/c mice." International Journal of Antimicrobial Agents.
68. Goncalves-Oliveira, L. F., et al. (2019). "The combination therapy of meglumine antimoniate and oxiranes (epoxy-alpha-lapachone and epoxymethyl-lawsone) enhance the leishmanicidal effect in mice infected by Leishmania (Leishmania) amazonensis." Int J Parasitol Drugs Drug Resist 10: 101-108.
69. Guimaraes, E. T., et al. (2009). "Activity of physalins purified from Physalis angulata in in vitro and in vivo models of cutaneous leishmaniasis." J Antimicrob Chemother 64(1): 84-87.
70. Gupta, G., et al. (2015). "A Novel Sterol Isolated from a Plant Used by Mayan Traditional Healers Is Effective in Treatment of Visceral Leishmaniasis Caused by Leishmania donovani." ACS Infectious Diseases 1(10): 497-506.
71. Gupta, S., et al. (2015). "Nitroimidazo-oxazole compound DNDI-VL-2098: an orally effective preclinical drug candidate for the treatment of visceral leishmaniasis." J Antimicrob Chemother 70(2): 518-527.
72. Heidari-Kharaji, M., et al. (2016). "Enhanced paromomycin efficacy by solid lipid nanoparticle formulation against Leishmania in mice model." Parasite Immunol 38(10): 599-608.
73. Honda, P. A., et al. (2010). "Efficacy of components from leaves of Calophyllum brasiliense against Leishmania (Leishmania) amazonensis." Phytomedicine 17(5): 333-338.
74. Hooshyar, H., et al. (2014). "Therapeutic effect of Hedera helix alcoholic extract against Cutaneous Leishmaniasis caused by Leishmania major in Balb/C mice." Jundishapur Journal of Microbiology 7.
75. Iman, M., et al. (2017). "Biodistribution and In Vivo Antileishmanial Activity of 1,2-Distigmasterylhemisuccinoyl-sn-Glycero-3-Phosphocholine Liposome-Intercalated Amphotericin B." Antimicrob Agents Chemother 61(9).
76. Inacio, J. D. F., et al. (2019). "(-)-Epigallocatechin 3- O-Gallate as a New Approach for the Treatment of Visceral Leishmaniasis." Journal of Natural Products 82(9): 2664-2667.
77. Iniguez, E., et al. (2016). "Ruthenium-Clotrimazole complex has significant efficacy in the murine model of cutaneous leishmaniasis." Acta Trop 164: 402-410.
78. Jaafari, M. R., et al. (2019). "Development of a topical liposomal formulation of Amphotericin B for the treatment of cutaneous leishmaniasis." International Journal for Parasitology: Drugs and Drug Resistance 11: 156-165.
79. Jesus, J. A., et al. (2017). "Therapeutic effect of ursolic acid in experimental visceral leishmaniasis." Int J Parasitol Drugs Drug Resist 7(1): 1-11.

80. Kansal, S., et al. (2012). "Development of nanocapsules bearing doxorubicin for macrophage targeting through the phosphatidylserine ligand: a system for intervention in visceral leishmaniasis." J Antimicrob Chemother 67(11): 2650-2660.
81. Kato, K. C., et al. (2014). "Hepatotoxicity of pentavalent antimonial drug: Possible role of residual sb(3) and protective effect of ascorbic acid." Antimicrobial Agents and Chemotherapy 58(1): 481-488.
82. Kaur, R., et al. (2016). "Evaluation of the antileishmanial efficacy of medicinal plant Chenopodium Album linn. against experimental visceral leishmaniasis." International Journal of Pharmacy and Pharmaceutical Sciences 8(7): 227-231.
83. Khadir, F., et al. (2019). "Antileishmanial effect of rapamycin as an alternative approach to control Leishmania tropica infection." Vet Parasitol 276: 108976.
84. Khayeka-Wandabwa, C., et al. (2013). "Combination therapy using Pentostam and Praziquantel improves lesion healing and parasite resolution in BALB/c mice co-infected with Leishmania major and Schistosoma mansoni." Parasit Vectors 6: 244.
85. Kheirandish, F., et al. (2016). "Antileishmanial, antioxidant, and cytotoxic activities of Quercus infectoria Olivier extract." Biomed Pharmacother 82: 208-215.
86. Khouri, R., et al. (2010). "DETC induces Leishmania parasite killing in human in vitro and murine in vivo models: a promising therapeutic alternative in Leishmaniasis." PLoS One 5(12): e14394.
87. Kuckelhaus, C. S., et al. (2011). "Influence of long-term treatment with pravastatin on the survival, evolution of cutaneous lesion and weight of animals infected by Leishmania amazonensis." Exp Parasitol 127(3): 658-664.
88. Kwofie, K. D., et al. (2019). "Oral activity of the antimalarial endoperoxide 6-(1,2,6,7-tetraoxaspiro[7.11]nonadec-4-yl)hexan-1-ol (N-251) against Leishmania donovani complex." PLoS Negl Trop Dis 13(3): e0007235.
89. Kyriazis, J. D., et al. (2013). "Leishmanicidal activity assessment of olive tree extracts." Phytomedicine 20(3): 275-281.
90. Laban, L. T., et al. (2015). "Experimental therapeutic studies of Solanum aculeastrum Dunal. On Leishmania major infection in BALB/c mice." Iranian Journal of Basic Medical Sciences 18(1): 64-71.
91. Lage, L. M., et al. (2016). "An 8-hydroxyquinoline-containing polymeric micelle system is effective for the treatment of murine tegumentary leishmaniasis." Parasitol Res 115(11): 4083-4095.
92. Lanza, J. S., et al. (2016). "Polarity-sensitive nanocarrier for oral delivery of Sb(V) and treatment of cutaneous leishmaniasis." Int J Nanomedicine 11: 2305-2318.
93. Lima, C. B., et al. (2009). "A novel organotellurium compound (RT-01) as a new antileishmanial agent." Korean J Parasitol 47(3): 213-218.
94. Machado, P. A., et al. (2017). "VOSalophen: a vanadium complex with a stilbene derivative-induction of apoptosis, autophagy, and efficiency in experimental cutaneous leishmaniasis." J Biol Inorg Chem 22(6): 929-939.
95. Machin, L., et al. (2019). "Bixa orellana L. (Bixaceae) and Dysphania ambrosioides (L.) Mosyakin & Clemants (Amaranthaceae) Essential Oils Formulated in Nanocochleates against Leishmania amazonensis." Molecules 24(23).
96. Mahmoudvand, H., et al. (2016). "In Vitro and In Vivo Antileishmanial Activities of Pistacia vera Essential Oil." Planta Med 82(4): 279-284.
97. Makwali, J. A., et al. (2012). "Combination and monotherapy of Leishmania major infection in BALB/c mice using plant extracts and herbicides." J Vector Borne Dis 49(3): 123-130.
98. Maleki, F., et al. (2017). "In vitro and in vivo susceptibility of Leishmania major to some medicinal plants." Asian Pacific Journal of Tropical Biomedicine 7(1): 37-42.
99. Malli, S., et al. (2019). "Combination of amphotericin B and chitosan platelets for the treatment of experimental cutaneous leishmaniasis: Histological and immunohistochemical examinations." Journal of Drug Delivery Science and Technology 50: 34-41.
100. Manhas, R., et al. (2018). "Leishmania donovani Parasites Are Inhibited by the Benzoxaborole AN2690 Targeting Leucyl-tRNA Synthetase." Antimicrob Agents Chemother 62(9).

101. Manzano, J. I., et al. (2019). "Discovery and pharmacological studies of 4-hydroxyphenyl-derived phosphonium salts active in a mouse model of visceral leishmaniasis." Journal of medicinal chemistry. 8.
102. Marango, S. N., et al. (2017). "Experimental therapeutic assays of Tephrosia vogelii against Leishmania major infection in murine model: in vitro and in vivo." BMC Res Notes 10(1): 698.
103. Masic, A., et al. (2015). "Cinnamic Acid Bornyl Ester Derivatives from Valeriana wallichii Exhibit Antileishmanial In Vivo Activity in Leishmania major-Infected BALB/c Mice." PLoS One 10(11): e0142386.
104. Mehrizi, T. Z., et al. (2018). "Novel nano-sized chitosan amphotericin B formulation with considerable improvement against Leishmania major." Nanomedicine (Lond) 13(24): 3129-3147.
105. Mendonca, D. V., et al. (2016). "Poloxamer 407 (Pluronic((R)) F127)-based polymeric micelles for amphotericin B: In vitro biological activity, toxicity and in vivo therapeutic efficacy against murine tegumentary leishmaniasis." Exp Parasitol 169: 34-42.
106. Mendonca, D. V. C., et al. (2018). "Comparing the therapeutic efficacy of different amphotericin B-carrying delivery systems against visceral leishmaniasis." Exp Parasitol 186: 24-35.
107. Mendonca, D. V. C., et al. (2019). "In vivo antileishmanial efficacy of a naphthoquinone derivate incorporated into a Pluronic((R)) F127-based polymeric micelle system against Leishmania amazonensis infection." Biomed Pharmacother 109: 779-787.
108. Mirzavand, S., et al. (2019). "In vitro and in vivo assessment of anti-leishmanial efficacy of leaf, fruit, and fractions of juniperus excelsa against axenic amastigotes of leishmania major and topical formulation in BALB/C mice." Iranian Red Crescent Medical Journal 21.
109. Mishra, J., et al. (2013). "Evaluation of toxicity & therapeutic efficacy of a new liposomal formulation of amphotericin B in a mouse model." Indian J Med Res 137(4): 767-776.
110. Misra, P., et al. (2010). "16alpha-Hydroxycleroda-3,13 (14)Z-dien-15,16-olide from Polyalthia longifolia: a safe and orally active antileishmanial agent." Br J Pharmacol 159(5): 1143-1150.
111. Mitra, M., et al. (2005). "Targeting of mannosylated liposome incorporated benzyl derivative of Penicillium nigricans derived compound MT81 to reticuloendothelial systems for the treatment of visceral leishmaniasis." J Drug Target 13(5): 285-293.
112. Mohebali, M., et al. (2009). "Nanosilver in the treatment of localized cutaneous leishmaniasis caused by Leishmania major (MRHO/IR/75/ER): An in vitro and in vivo study." Daru 17(4): 285-289.
113. Montrieux, E., et al. (2014). "In vitro and in vivo activity of major constituents from Pluchea carolinensis against Leishmania amazonensis." Parasitol Res 113(8): 2925-2932.
114. Monzote, L., et al. (2016). "In-vitro and in-vivo activities of phenolic compounds against cutaneous leishmaniasis." Records of Natural Products 10(3): 269-276.
115. Morais-Teixeira, E. d., et al. (2008). "In vitro and in vivo activity of meglumine antimoniate produced at Farmanguinhos-Fiocruz, Brazil, against Leishmania (Leishmania) amazonensis, L (L) chagasi and L (Viannia) braziliensis^ien." Mem. Inst. Oswaldo Cruz 103(4): 358-362.
116. Moreno, E., et al. (2019). "Evaluation of skin permeation and retention of topical dapsone in murine cutaneous leishmaniasis lesions." Pharmaceutics 11(607).
117. Mostafavi, M., et al. (2019). "Niosomal formulation of amphotericin B alone and in combination with glucantime: In vitro and in vivo leishmanicidal effects." Biomed Pharmacother 116: 108942.
118. Mowbray, C. E., et al. (2015). "Novel Amino-pyrazole Ureas with Potent In Vitro and In Vivo Antileishmanial Activity." J Med Chem 58(24): 9615-9624.
119. Muñoz, B. Y., et al. (2019). "Therapeutic response and safety of the topical, sequential use of antiseptic, keratolytic, and pentamidine creams (3-PACK) on Leishmania (Viannia) braziliensis-infected mice^ien." Mem. Inst. Oswaldo Cruz 114.
120. Muzitano, M. F., et al. (2009). "Oral metabolism and efficacy of Kalanchoe pinnata flavonoids in a murine model of cutaneous leishmaniasis." Planta Med 75(4): 307-311.
121. Nahrevanian, H., et al. (2007). "Pharmacological evaluation of anti-leishmanial activity by in vivo nitric oxide modulation in Balb/c mice infected with Leishmania major MRHO/IR/75/ER: an Iranian strain of cutaneous leishmaniasis." Exp Parasitol 116(3): 233-240.
122. Nahrevanian, H., et al. (2009). "Anti-leishmanial effects of trinitroglycerin in BALB/C mice infected with Leishmania major via nitric oxide pathway." Korean J Parasitol 47(2): 109-115.

123. Naman, C. B., et al. (2015). "Northalrugosidine is a bisbenzyltetrahydroisoquinoline alkaloid from Thalictrum alpinum with in vivo antileishmanial activity." J Nat Prod 78(3): 552-556.
124. Nandan, D., et al. (2018). "Miransertib (ARQ 092), an orally-available, selective Akt inhibitor is effective against Leishmania." PLoS One 13(11): e0206920.
125. Nascimento, K. F., et al. (2017). "M1 homeopathic complex trigger effective responses against Leishmania (L) amazonensis in vivo and in vitro." Cytokine 99: 80-90.
126. Nascimento, N., et al. (2019). "In vitro and in vivo leishmanicidal activity of a ruthenium nitrosyl complex against Leishmania (Viannia) braziliensis." Acta Trop 192: 61-65.
127. Nieto-Yanez, O. J., et al. (2017). "In vivo and in vitro antileishmanial effects of methanolic extract from of Bursera aptera." Afr J Tradit Complement Altern Med 14(2): 188-197.
128. Oliveira, L. F. G., et al. (2018). "Antileishmanial Activity of 2-Methoxy-4H-spiro-[naphthalene-1,2'-oxiran]-4-one (Epoxymethoxy-lawsone): A Promising New Drug Candidate for Leishmaniasis Treatment." Molecules 23(4).
129. Oryan, A., et al. (2018). "Efficacy of voriconazole on leishmaniasis by Leishmania major: An in vitro and in vivo study." Asian Pacific Journal of Tropical Medicine 11(10): 562-569.
130. Ozbilgin, A., et al. (2014). "Antileishmanial activity of selected Turkish medicinal plants." Tropical Journal of Pharmaceutical Research 13(12): 2047-2055.
131. Paik, D., et al. (2016). "Protective inflammatory response against visceral leishmaniasis with potato tuber extract: A new approach of successful therapy." Biomed Pharmacother 83: 1295-1302.
132. Paladi Cde, S., et al. (2012). "In vitro and in vivo activity of a palladacycle complex on Leishmania (Leishmania) amazonensis." PLoS Negl Trop Dis 6(5): e1626.
133. Palit, P., et al. (2012). "Discovery of safe and orally effective 4-aminoquinaldine analogues as apoptotic inducers with activity against experimental visceral leishmaniasis." Antimicrob Agents Chemother 56(1): 432-445.
134. Pandey, S., et al. (2016). "Identification of a diverse indole-2-carboxamides as a potent antileishmanial chemotypes." Eur J Med Chem 110: 237-245.
135. Patricio, F. J., et al. (2008). "Efficacy of the intralesional treatment with Chenopodium ambrosioides in the murine infection by Leishmania amazonensis." J Ethnopharmacol 115(2): 313-319.
136. Poddar, A., et al. (2008). "In vivo efficacy of calceolarioside A against experimental visceral leishmaniasis." Planta Med 74(5): 503-508.
137. Prajapati, V. K., et al. (2011). "Targeted killing of Leishmania donovani in vivo and in vitro with amphotericin B attached to functionalized carbon nanotubes." J Antimicrob Chemother 66(4): 874-879.
138. Prajapati, V. K., et al. (2012). "An oral formulation of amphotericin B attached to functionalized carbon nanotubes is an effective treatment for experimental visceral leishmaniasis." J Infect Dis 205(2): 333-336.
139. Queiroz, D. P. d. S., et al. (2016). "In vivo antileishmanial activity and chemical profile of polar extract from Selaginella sellowii^ien." Mem. Inst. Oswaldo Cruz 111(3): 147-154.
140. Rabito, M. F., et al. (2014). "In vitro and in vivo antileishmania activity of sesquiterpene lactone-rich dichloromethane fraction obtained from Tanacetum parthenium (L.) Schultz-Bip." Exp Parasitol 143: 18-23.
141. Rafiee, A., et al. (2014). "Ferroportin-encapsulated nanoparticles reduce infection and improve immunity in mice infected with Leishmania major." Int J Pharm 466(1): 375-381.
142. Rahimi-Moghaddam, P., et al. (2011). "In vitro and in vivo activities of peganum harmala extract against leishmania major." Journal of Research in Medical Sciences 16(8).
143. Rastegarian, M., et al. (2019). "In vivo assay of wound healing activities of silymarin extract on cutaneous wounds caused by Leishmania major." Shiraz E Medical Journal 20.
144. Rathore, A., et al. (2011). "Mannosylated liposomes bearing Amphotericin B for effective management of visceral Leishmaniasis." J Liposome Res 21(4): 333-340.
145. Ravichandran, V., et al. (2018). "New Water-Soluble Polymeric Prodrugs of 2-n-propylquinoline: Synthesis and Evaluation of In Vitro and In Vivo Activities Against Leishmania donovani." Regenerative Engineering and Translational Medicine 4(1): 11-20.

146. Ray, L., et al. (2020). "Efficient antileishmanial activity of amphotericin B and piperine entrapped in enteric coated guar gum nanoparticles." Drug Delivery and Translational Research.
147. Rebello, K. M., et al. (2019). "Miltefosine-Lopinavir Combination Therapy Against Leishmania infantum Infection: In vitro and in vivo Approaches." Front Cell Infect Microbiol 9: 229.
148. Reimao, J. Q., et al. (2014). "Antileishmanial activity of the estrogen receptor modulator raloxifene." PLoS Negl Trop Dis 8(5): e2842.
149. Reis, L. E. S., et al. (2017). "Mixed Formulation of Conventional and Pegylated Meglumine Antimoniate-Containing Liposomes Reduces Inflammatory Process and Parasite Burden in Leishmania infantum-Infected BALB/c Mice." Antimicrob Agents Chemother 61(11).
150. Ribeiro, J. B. P., et al. (2019). "Study of the efficacy of N-methyl glucamine antimoniate (Sb(V)) associated with photodynamic therapy using liposomal chloroaluminium phthalocyanine in the treatment of cutaneous leishmaniasis caused by Leishmania (L.) amazonensis in C57BL6 mice." Photodiagnosis Photodyn Ther 26: 261-269.
151. Ribeiro, T. G., et al. (2014). "An optimized nanoparticle delivery system based on chitosan and chondroitin sulfate molecules reduces the toxicity of amphotericin B and is effective in treating tegumentary leishmaniasis." Int J Nanomedicine 9: 5341-5353.
152. Rice, D. R., et al. (2016). "Zinc(II)-Dipicolylamine Coordination Complexes as Targeting and Chemotherapeutic Agents for Leishmania major." Antimicrob Agents Chemother 60(5): 2932-2940.
153. Robledo, S. M., et al. (2018). "Arnica Tincture Cures Cutaneous Leishmaniasis in Golden Hamsters." Molecules 23(1).
154. Rodrigues, A. P., et al. (2014). "A novel function for kojic acid, a secondary metabolite from Aspergillus fungi, as antileishmanial agent." PLoS One 9(3): e91259.
155. Rodrigues, K., et al. (2018). "SB-83, a 2-Amino-thiophene derivative orally bioavailable candidate for the leishmaniasis treatment." Biomed Pharmacother 108: 1670-1678.
156. Roy, S., et al. (2017). "Mahanine exerts in vitro and in vivo antileishmanial activity by modulation of redox homeostasis." Scientific reports 7(1): 4141.
157. Roychoudhury, J., et al. (2011). "Therapy with sodium stibogluconate in stearylamine-bearing liposomes confers cure against SSG-resistant Leishmania donovani in BALB/c mice." PLoS One 6(3): e17376.
158. Saha, S., et al. (2016). "A Novel Spirooxindole Derivative Inhibits the Growth of Leishmania donovani Parasites both In Vitro and In Vivo by Targeting Type IB Topoisomerase." Antimicrob Agents Chemother 60(10): 6281-6293.
159. Saha, S., et al. (2013). "The lignan glycosides lyoniside and saracoside poison the unusual type IB topoisomerase of Leishmania donovani and kill the parasite both in vitro and in vivo." Biochem Pharmacol 86(12): 1673-1687.
160. Salerno Pimentel, I. A., et al. (2012). "In vitro and in vivo activity of an organic tellurium compound on Leishmania (Leishmania) chagasi." PLoS One 7(11): e48780.
161. Santos, D., et al. (2018). "A new nanoemulsion formulation improves antileishmanial activity and reduces toxicity of amphotericin B." J Drug Target 26(4): 357-364.
162. Santos, D. M., et al. (2014). "Chemotherapeutic potential of 17-AAG against cutaneous leishmaniasis caused by Leishmania (Viannia) braziliensis." PLoS Negl Trop Dis 8(10): e3275.
163. Scariot, D. B., et al. (2019). "Oral treatment with T6-loaded yeast cell wall particles reduces the parasitemia in murine visceral leishmaniasis model." Scientific reports 9(1): 20080.
164. Sen, R., et al. (2010). "Efficacy of artemisinin in experimental visceral leishmaniasis." Int J Antimicrob Agents 36(1): 43-49.
165. Serna, M. E., et al. (2015). "Finding of leishmanicidal activity of 14-hydroxylunularin in mice experimentally infected with Leishmania infantum." Parasitol Int 64(5): 295-298.
166. Sghaier, R. M., et al. (2016). "Treatment with synthetic lipophilic tyrosyl ester controls Leishmania major infection by reducing parasite load in BALB/c mice." Parasitology 143(12): 1615-1621.
167. Sharifi-Rad, J., et al. (2018). "Susceptibility of Leishmania major to Veronica persica Poir. extracts - In vitro and in vivo assays." Cell Mol Biol (Noisy-le-grand) 64(8): 44-49.

168. Sharlow, E. R., et al. (2009). "Identification of potent chemotypes targeting Leishmania major using a high-throughput, low-stringency, computationally enhanced, small molecule screen." PLoS Neglected Tropical Diseases 3.
169. Shio, M. T., et al. (2013). "Drug delivery by tattooing to treat cutaneous leishmaniasis." Tropical Medicine and International Health 1: 60-61.
170. Sobarzo-Sanchez, E., et al. (2013). "Synthetic oxoisoaporphine alkaloids: in vitro, in vivo and in silico assessment of antileishmanial activities." PLoS One 8(10): e77560.
171. Sousa, J. K. T., et al. (2019). "A chloroquinoline derivate presents effective in vitro and in vivo antileishmanial activity against Leishmania species that cause tegumentary and visceral leishmaniasis." Parasitol Int 73: 101966.
172. Sousa-Batista, A. J., et al. (2018). "Broad Spectrum and Safety of Oral Treatment with a Promising Nitrosylated Chalcone in Murine Leishmaniasis." Antimicrob Agents Chemother 62(10).
173. Sousa-Batista, A. J., et al. (2018). "Depot Subcutaneous Injection with Chalcone CH8-Loaded Poly(Lactic-Co-Glycolic Acid) Microspheres as a Single-Dose Treatment of Cutaneous Leishmaniasis." Antimicrob Agents Chemother 62(3).
174. Sousa-Batista, A. J., et al. (2019). "Novel and safe single-dose treatment of cutaneous leishmaniasis with implantable amphotericin B-loaded microparticles." International Journal for Parasitology: Drugs and Drug Resistance 11: 148-155.
175. Sousa-Batista, A. J., et al. (2017). "Lipid-core nanocapsules increase the oral efficacy of quercetin in cutaneous leishmaniasis." Parasitology 144(13): 1769-1774.
176. Souza-Silva, F., et al. (2015). "Epoxy-alpha-lapachone has in vitro and in vivo anti-leishmania (Leishmania) amazonensis effects and inhibits serine proteinase activity in this parasite." Antimicrob Agents Chemother 59(4): 1910-1918.
177. Soyer, T. G., et al. (2019). "Evaluation of the in vitro and in vivo antileishmanial activity of a chloroquinolin derivative against Leishmania species capable of causing tegumentary and visceral leishmaniasis." Exp Parasitol 199: 30-37.
178. Tavares, G. S. V., et al. (2019). "In vitro and in vivo antileishmanial activity of a fluoroquinoline derivate against Leishmania infantum and Leishmania amazonensis species." Acta Trop 191: 29-37.
179. Tavares, G. S. V., et al. (2019). "A Pluronic(R) F127-based polymeric micelle system containing an antileishmanial molecule is immunotherapeutic and effective in the treatment against Leishmania amazonensis infection." Parasitol Int 68(1): 63-72.
180. Teixeira, M. J., et al. (2001). "In vitro and in vivo Leishmanicidal activity of 2-hydroxy-3-(3-methyl-2-butenyl)-1,4-naphthoquinone (lapachol)." Phytother Res 15(1): 44-48.
181. Tempone, A. G., et al. (2010). "Therapeutic evaluation of free and liposome-loaded furazolidone in experimental visceral leishmaniasis." Int J Antimicrob Agents 36(2): 159-163.
182. Thorstenberg, M. L., et al. (2018). "Purinergic cooperation between P2Y2 and P2X7 receptors promote cutaneous leishmaniasis control: Involvement of pannexin-1 and leukotrienes." Frontiers in Immunology 9(1531).
183. Tiwari, B., et al. (2017). "Nanotized Curcumin and Miltefosine, a Potential Combination for Treatment of Experimental Visceral Leishmaniasis." Antimicrob Agents Chemother 61(3).
184. Trinconi, C. T., et al. (2018). "Topical tamoxifen in the therapy of cutaneous leishmaniasis." Parasitology 145(4): 490-496.
185. Trinconi, C. T., et al. (2016). "Efficacy of tamoxifen and miltefosine combined therapy for cutaneous leishmaniasis in the murine model of infection with Leishmania amazonensis." J Antimicrob Chemother 71(5): 1314-1322.
186. Trinconi, C. T., et al. (2014). "Combination therapy with tamoxifen and amphotericin B in experimental cutaneous leishmaniasis." Antimicrob Agents Chemother 58(5): 2608-2613.
187. Upadhyay, A., et al. (2019). "Synthesis, Biological Evaluation, Structure-Activity Relationship, and Mechanism of Action Studies of Quinoline-Metronidazole Derivatives Against Experimental Visceral Leishmaniasis." Journal of Medicinal Chemistry 62(11): 5655-5671.
188. Valadares, D. G., et al. (2012). "Therapeutic efficacy induced by the oral administration of Agaricus blazei Murill against Leishmania amazonensis." Parasitol Res 111(4): 1807-1816.

189. Valdivieso, E., et al. (2018). "Potentiation of the leishmanicidal activity of nelfinavir in combination with miltefosine or amphotericin B." Int J Antimicrob Agents 52(5): 682-687.
190. Van Bocxlaer, K., et al. (2016). "Topical formulations of miltefosine for cutaneous leishmaniasis in a BALB/c mouse model." J Pharm Pharmacol 68(7): 862-872.
191. Van den Kerkhof, M., et al. (2018). "In vitro and in vivo pharmacodynamics of three novel antileishmanial lead series." Int J Parasitol Drugs Drug Resist 8(1): 81-86.
192. Varela, M. R., et al. (2012). "In vitro and in vivo efficacy of ether lipid edelfosine against Leishmania spp. and SbV-resistant parasites." PLoS Negl Trop Dis 6(4): e1612.
193. Varikuti, S., et al. (2019). "The Potent ITK/BTK Inhibitor Ibrutinib Is Effective for the Treatment of Experimental Visceral Leishmaniasis Caused by Leishmania donovani." J Infect Dis 219(4): 599-608.
194. Veerareddy, P. R., et al. (2009). "Antileishmanial activity, pharmacokinetics and tissue distribution studies of mannose-grafted amphotericin B lipid nanospheres." J Drug Target 17(2): 140-147.
195. Velasquez, A. M. A., et al. (2017). "Efficacy of a Binuclear Cyclopalladated Compound Therapy for Cutaneous Leishmaniasis in the Murine Model of Infection with Leishmania amazonensis and Its Inhibitory Effect on Topoisomerase 1B." Antimicrob Agents Chemother 61(8). dentifier NCT02169141.).
196. Villa-Pulgarin, J. A., et al. (2017). "Mitochondria and lipid raft-located ATP synthase as major therapeutic targets in the antileishmanial and anticancer activities of ether lipid edelfosine." PLoS Neglected Tropical Diseases 11.
197. Want, M. Y., et al. (2017). "Nanoliposomal artemisinin for the treatment of murine visceral leishmaniasis." Int J Nanomedicine 12: 2189-2204.
198. Wijnant, G. J., et al. (2018). "Comparative efficacy, toxicity and biodistribution of the liposomal amphotericin B formulations Fungisome((R)) and AmBisome((R)) in murine cutaneous leishmaniasis." Int J Parasitol Drugs Drug Resist 8(2): 223-228.
199. Wijnant, G. J., et al. (2018). "Relation between Skin Pharmacokinetics and Efficacy in AmBisome Treatment of Murine Cutaneous Leishmaniasis." Antimicrob Agents Chemother 62(3).
200. Wijnant, G. J., et al. (2017). "Efficacy of Paromomycin-Chloroquine Combination Therapy in Experimental Cutaneous Leishmaniasis." Antimicrob Agents Chemother 61(8).
201. Wong, I. L., et al. (2014). "In vitro and in vivo efficacy of novel flavonoid dimers against cutaneous leishmaniasis." Antimicrob Agents Chemother 58(6): 3379-3388.
202. Youssefi, M. R., et al. (2019). "In Vitro and In Vivo Effectiveness of Carvacrol, Thymol and Linalool against Leishmania infantum." Molecules 24(11).
203. Zahedifard, F., et al. (2019). "Anti-leishmanial activity of Brevinin 2R and its Lauric acid conjugate type against L. major: In vitro mechanism of actions and in vivo treatment potentials." PLoS Negl Trop Dis 13(2): e0007217.
